# Design and Evaluation of Voriconazole Loaded Solid Lipid Nanoparticles for Ophthalmic Application

**DOI:** 10.1155/2016/6590361

**Published:** 2016-05-12

**Authors:** Anubha Khare, Inderbir Singh, Pravin Pawar, Kanchan Grover

**Affiliations:** ^1^Chitkara College of Pharmacy, Chitkara University, Chandigarh-Patiala National Highway, Rajpura, Patiala, Punjab 140401, India; ^2^Gourishankar Institute of Pharmaceutical Education & Research, Limb, Satara, Survey no. 990, Near NH-4, Satara 415020, India

## Abstract

Voriconazole is a second-generation antifungal agent with excellent broad spectrum of antifungal activity commercially available for oral and intravenous administration. Systemic administration of voriconazole is associated with side effects including visual and hepatic abnormalities. This study assessed the feasibility of using solid lipid nanoparticles for ocular delivery of voriconazole adopting stearic acid as lipidic material, tween 80 as a stabilizer, and Carbopol 934 as controlled release agent and for increasing the precorneal residence time in eye. The systems were prepared using two different methods, that is, ultrasonication method and microemulsion technique. The results indicated that the larger particle size of SLNs was found with microemulsion technique (308 ± 3.52 nm to 343 ± 3.51) compared to SLN prepared with ultrasonication method (234 ± 3.52 nm to 288 ± 4.58 nm). The polydispersity index values were less than 0.3 for all formulations and zeta potential of the prepared formulations by these two methods varied from −22.71 ± 0.63 mV to −28.86 ± 0.58 mV. Powder X-ray diffraction and differential scanning calorimetry indicated decrease in crystallinity of drug. The* in vitro* release study and the SLN formulations prepared with ultrasonication method demonstrated sustained release up to 12 hours. This study demonstrated that SLN prepared by ultrasonication method is more suitable than microemulsion technique without causing any significant effect on corneal hydration level.

## 1. Introduction

Eye is the most simply accessible site for topical administration of a medication. Drugs are commonly applied to the ocular system for a localized action on the surface or in the interior of the eye [1]. The major challenge to the formulator is to outwit these barriers without causing any tissue damage [2]. The cornea is the anterior layer of the eye, comprised of epithelium, stroma, and endothelium. However, this layer represents a mechanical barrier restraining the delivery of drug molecules. Due to their high lipid content, the epithelium and the endothelium are considered as an obstruction to the passage of hydrophilic molecules. The stroma is characterized by a high water content that makes this layer impermeable to lipophilic molecules [3]. Corneal barrier also plays a considerable role in low ocular bioavailability due to which only <5% of the applied drugs are able to penetrate through the cornea into the intraocular tissues [4].

As a result to optimize ocular drug delivery, different strategies were developed to increase the bioavailability of drugs in the front of eye to prolong time. An ideal ophthalmic drug delivery must be able to release the drug in sustained manner and remain in the front of eye to prolong period of the time. As a result various attempts have been made to prolong the residence time of drug on the ocular surface and also to slow down the drug elimination [5, 6].

To overcome the above problems related to ocular delivery, novel drug delivery systems (NDDS) consisting mainly of nanoemulsions, liposomes, microemulsions, microspheres, and solid lipid nanoparticles (SLNs) have been projected newly for oral, topical, and parental administration of drugs [6, 7]. SLN represented an exceptional carrier system to conventional colloidal carriers, such as emulsions, lipid emulsion, liposomes, and polymeric microparticles, and so forth. SLNs are submicron colloidal carriers ranging from 50 to 1000 nm, which are composed of physiological lipid, dispersed in water or in aqueous surfactant solution [8]. They are appropriate for the incorporation of lipophilic, hydrophilic, and poorly water soluble drugs [9]. Various researchers have been working in the area of ocular drug delivery; that is, formulated meloxicam solid lipid nanoparticles were formulated and it was concluded that SLNs have excellent physical stability, high entrapment efficiency, and sustained drug release and also represent a promising carrier for topical delivery of meloxicam [10]. Some researchers describe the preparation and characterization of solid lipid nanoparticles (SLNs) prepared with stearic acid (SLN-A) and a mixture of stearic acid and Compritol (SLN-B) as lipid matrix and poloxamer-188 as surfactant, using sodium taurocholate and ethanol as cosurfactant mixture, which proves SLN to be a good ocular drug delivery system considering the smaller particle size, particle size stability, physiologically tolerable components, and so forth [9]. Amphotericin B and natamycin fungal ulcers tend to have very poor outcomes. Since 1960 no new medications have been permitted by the FDA and there has been only a randomized trial of antifungal therapy for fungal ulcers [11]. There are studies that indicate the newer triazoles; that is, voriconazole is a novel second-generation triazole derivative of fluconazole with exceptional broad spectrum activity commercially available for oral and intravenous administration. Voriconazole has broad* in vitro* antifungal activity against yeasts and moulds, including a broad range of less common pathogens. Voriconazole possesses fungicidal* in vitro* activity against all* Aspergillus* species, moulds such as* Scedosporium* species and* Fusarium* species, and is highly potent against fluconazole-resistant* Candida* species including* Candida krusei*,* Candida glabrata*, and* Candida albicans*. The minimum inhibitory concentrations (MIC90) of voriconazole are considerably less than that of fluconazole. It inhibits cytochrome P450-dependent 14*α* sterol demethylase enzyme which is responsible for inhibiting and disrupting fungal cell membrane synthesis, resulting in depletion of ergosterol [12]. Voriconazole marketed formulations are also present; namely, Vozole is a lyophilized powder (VCZ eye drops 1% w/v and VCZ injection 1 mg/vial).

Voriconazole is a lipophilic drug with a low pH dependent aqueous solubility (maximum 2.7 mg/mL at pH 1.2). To improve the accessibility of voriconazole at the intraocular level, voriconazole loaded solid lipid formulations were prepared. Stearic acid is a saturated, wax-like, fatty acid commonly used in the production of SLNs. In addition to this, it has excellent biodegradability and has low toxicity and also incorporates both hydrophilic and lipophilic drugs [13]. Tween 80 is a nonionic surfactant derived from sorbitan esters and acting as stabilizer in the preparation [14]. Carbopol 934 is a polyacrylic polymer which has inherent mucoadhesiveness, acts as controlled release agent, and also prolongs the precorneal residence time and avoids systemic side effects [15].

The purpose of the study was to formulate voriconazole loaded-SLN formulations as sustained ocular drug delivery systems with the aim of improving availability of voriconazole at the intraocular level. It was proposed to prepare the SLN of voriconazole by both ultrasonication and microemulsion techniques so that the best suitable method out of these two can be projected and to determine the effect of formulation factors on the transcorneal permeation of voriconazole through freshly excised goat corneas.

## 2. Materials and Methods

### 2.1. Materials

Voriconazole was received as gift samples from Matrix Laboratories, Hyderabad (India), and stearic acid and Carbopol 934 were purchased from Sigma-Aldrich Pvt. Ltd. (India). All other chemicals used were of analytical reagent grade. Fresh whole eyeballs of goat were obtained from local butcher's shop (Zirakpur, Punjab, India) within one hour of slaughtering of animal.

### 2.2. Methods

#### 2.2.1. Ultrasonication Method

Firstly, stearic acid was melted in porcelain dish (for relaxation of lipid side chain) and then required quantity of tween 80 was added. Secondly, drug was soluble in dichloromethane and this drug solution was added in the above lipid phase. The detailed formulae of VCZ loaded SLNs with their percentage (w/w or w/v) are given in Table 1. An aqueous phase containing different concentrations of Carbopol 934 was prepared in 20 mL of distilled water with constant stirring and heating at approximately 70°C in which lipid phase is added slowly. The prepared solution was stirred at 2000 rpm for 3 min and instantly ultrasonicated by probe sonicator (PCI analytics, Mumbai, India). Ultrasonication was done by adjusting frequency 0.5 for 30 min at 45% amplitude. After sonication, the dispersion was diluted with 80 mL of distilled cold water under continuous stirring for 15 min. A stable SLN suspension of voriconazole by ultrasonication (VUSLN) was obtained [16].

#### 2.2.2. Microemulsion Technique

In this technique, stearic acid was melted in porcelain dish (for relaxation of lipid side chain) and then required quantity of tween 80 was added. Then, drug was soluble in dichloromethane and this drug solution was added in the above lipid phase. The detailed formulae of VCZ loaded SLNs with their percentage (w/w or w/v) are given in Table 1. An aqueous phase containing different concentrations of Carbopol 934 was prepared in 20 mL of distilled water in which lipid phase is added slowly with constant stirring and heating at approximately 70°C for 15 minutes. The solid lipid nanoparticles were obtained by dispersing warm o/w microemulsion dropwise into cold water (2-3°C) in a beaker under continuous stirring at 2000 rpm for 4 hr. After completion of stirring, SLN dispersion was instantly ultrasonicated by probe sonicator (PCI analytics, Mumbai, India). Ultrasonication was done by adjusting frequency 0.5 for 30 min at 45% amplitude. A stable SLN suspension of voriconazole by microemulsion (VMSLN) technique was obtained [17].

#### 2.2.3. Lyophilization

To these above prepared aqueous dispersions, 2.5% w/v mannitol was dissolved as a cryoprotectant and then lyophilization was carried out for 24 hr to verify physical stability and redispersibility. Firstly, prefreezing was done by freezing the mixture at −74°C, at 0.02 mm Hg pressure, and then vials were kept in adapter. The adapter was then fit into freeze-dryer (Lyophilizer FD-5-3, Allied Frost, New Delhi, India) and dispersion was lyophilized in a laboratory to get free flowing powder of voriconazole SLN [18, 19].

## 3. Physicochemical Characterization of VCZ-SLN

### 3.1. Particle Size, Polydispersity Index, and Zeta Potential Measurements

The particle size and polydispersity index (PDI) of the SLN were determined by photon correlation spectroscopy (PCS) with a Zetasizer Nano ZS-90 (Malvern Instruments Ltd., Worcestershire, UK). Prior to analysis, samples of all SLN formulations were diluted with double distilled water. The zeta potential measurements were done by laser-doppler-anemometer coupled with Zetasizer Nano ZS-90 (Malvern Instruments Ltd., Worcestershire, UK) to validate the electrophoretic mobility of particles. All the analysis were repeated in triplicate.

### 3.2. Determination of Entrapped VCZ

It is the percentage of the actual mass of drug that was entrapped in the polymeric carrier, relative to the initial amount of loaded drug, and was calculated using the following equation:

(1) $$\begin{array}{c} \text{Entrapment efficiency}\%=\frac{\text{Actual loading}}{\text{Theoretical loading}}\times 100. \end{array}$$

Theoretical drug loading was calculated from the amount of drug taken relative to the amount of total drug and excipients used in the preparation of nanoparticles as follows:

(2) Theoretical  loading%=Total  drugTotal  drug+Total  excipients.

For actual drug loading, the SLN dispersion prepared by dispersing 25 mg of the lyophilized SLN powder in 5 mL mixture of methanol : distilled water (1 : 4) was centrifuged at 13000 rpm for 20 min. The clear supernatant was analyzed for free voriconazole content by measuring absorbance at 255 nm in an UV-visible spectrophotometer (Systronics, Mumbai). The total amount of drug present in the SLN was determined by dispersing 25 mg of the lyophilized SLN powder in 10 mL dichloromethane by sonication and filtering through a microsyringe filter (0.2 *μ*m) and analyzing the filtrate for voriconazole by measuring absorbance at 255 nm using UV-visible spectrophotometer [18]. The following formula was used to calculate actual loading:

(3) Actual  loading%=Total  drug−Free  drugmg  of  lyophilized  powder×100.

### 3.3. Fourier Transform Infrared Spectroscopy (FTIR) Analysis

FTIR spectra of prepared SLN were recorded by Bruker spectrophotometer (Bruker IFS 66/S, Germany), using the potassium bromide (KBr) disk technique (5 mg samples for 100 mg dry KBr). KBr discs of the lyophilized formulations were prepared and analyzed at the wavelength range of 4000–400 cm^−1^.

### 3.4. Electron Microscope Examination

The morphological observation of the VCZ loaded SLN was performed by transmission electron microscope (TEM). The samples of nanoparticles were stained with 2% (w/v) phosphotungstic acid. The nanoparticle suspension (5–10 *μ*L) was placed on the copper grids with films for viewing by TEM (Hitachi H-7500, Tokyo, Japan). Digital micrograph and imaging viewer software was used to capture the image.

### 3.5. Powder X-Ray Diffraction (PXRD) Analysis

PXRD was done to examine the crystalline state of the formulated SLN. The X-ray powder diffraction patterns of the samples were recorded with the XPERT-PRO multipurpose X-ray diffractometer (PAN analytical, Netherlands) using the PRS measurement program using Ni-filtered, CuK*α* radiation generated at 45 kV, and a current intensity of 40 mA. The diffraction angle range of the instrument was operated over a range of 2*θ* angles from 5° to 40°.

### 3.6. Differential Scanning Calorimetry (DSC) Analysis

Thermograms of the different samples were obtained using a DSC TA-60 (Shimadzu, Tokyo, Japan) 208 calorimeter. Samples (3–5 mg) were heated in crimped aluminum pans from 40°C to 200°C at a scanning rate of 10°C/min. Analyses were carried out under an inert nitrogen purge (35 mL/min) and an empty pan of alumina was used as reference in every case.

### 3.7. *In Vitro* Drug Release from VCZ-SLN

The* in vitro* drug release studies were carried out in the modified USP dissolution apparatus 1 (37 ± 0.5°C) containing a two-sided open glass cylinder for 12 hr. The diffusion barrier was dialysis membrane, and molecular weight cut-off 12000–14000 A (Himedia, Mumbai) was a release barrier. A presoaked dialysis membrane was adapted to the terminal portion of the glass cylinder. In each case, solid lipid nanosuspension (5 mL) was accurately introduced into the glass cylinder from the open side and this cylinder was fixed on the stirrer. The stirrer was suspended in 100 mL dissolution of simulated tear fluid (pH 7.4) medium maintained at 37°C ± 0.5°C at 100 rpm. Aliquots of samples were withdrawn at predetermined time intervals with volume replacement. The withdrawn samples were analyzed for drug content, by measuring absorbance at 255 nm in the UV-visible spectrophotometer (Systronics, Mumbai, India). Sink conditions were maintained throughout the release period. Data obtained in triplicate were analyzed graphically; that is, percent drug release versus time graph was plotted [14].

### 3.8. Release Kinetics

The kinetics of voriconazole release from solid lipid nanoparticles was determined using the release kinetics method of drug release into various kinetic equations: zero-order release kinetics, first-order release kinetics, and Higuchi model. The release data obtained was calculated using various parameters. The parameters “*n*” and time component “*k*,” the release rate constant, and “*R*” the regression coefficient were determined by Korsmeyer-Peppas equation to understand the release mechanism [20].

### 3.9. Corneal Permeation Studies

Drug permeation studies were carried out by putting the voriconazole solid lipid nanoparticles (5 mL) on a freshly excised goat cornea. The fresh, whole eyeballs of goats were obtained from a local butcher's shop and transported to the laboratory chilled in normal saline (4°C). The cornea was then carefully excised along with 2 to 4 mm of surrounding scleral tissue and was washed with normal saline until the washing was free from protein. The excised cornea was fixed between the clamped donor and receptor compartments of an all-glass modified Franz diffusion cell in such a way that its epithelial surface faced the donor compartment at a temperature of 37 ± 0.5°C. The corneal area available for diffusion was 0.75 cm^2^. The receptor compartment was filled with 10 mL freshly prepared simulated tear fluid (pH 7.4), and all air bubbles were expelled from the compartment. The whole corneal preparation procedure was completed within 1 hr after the sacrifice of goat. The aliquots (1 mL) of the formulated nanoparticles were placed on the excised cornea and the opening of the donor cell was sealed with a glass cover slip to prevent evaporation. The acceptor solution was kept at 37°C with constant stirring using a Teflon-coated magnetic stir bead. The permeation study was carried out for 120 min, and samples were withdrawn from the receptor at predetermined time intervals. The withdrawn samples were analyzed for drug content by measuring absorbance at 255 nm in a UV/Visible spectrophotometer (Systronics, Mumbai, India) [21, 22].

#### 3.9.1. Corneal Hydration Studies

At the end of the experiment, each cornea (freed from adhering sclera) was weighed, soaked in 1 mL methanol, dried overnight at 80°C, and reweighed. From the difference in weights, corneal hydration was calculated [21]:

(4) Corneal  hydration=Initial  weight−Final  weightInitial  weight×100.

### 3.10. Stability Assessment

Stability studies were performed on voriconazole loaded solid lipid nanoparticles according to ICH guidelines. All prepared formulations were stored in closed amber glass at room temperature conditions. Samples were withdrawn at time intervals 0 days, 3 weeks, 6 weeks, and 3 months. The samples were evaluated for their drug content, entrapment efficiency, and particle size. The degradation rate constant (*K*_cal_), shelf life, *t*_90_, and initial concentrations were also calculated.

### 3.11. Data Analysis

All values presented in the study are average of triplicate experiments for the same time points. Particle size, zeta potential, polydispersity index (PDI) entrapment efficiency,* in vitro* drug release, and* ex vivo* permeation profile of voriconazole SLN were tested statistically using one-way analysis of variance (ANOVA) followed by Dunnett's test using graph pad prism 5 software (GraphPad Software Inc., San Diego, CA) at different level of significance (^†^statistically significant difference at *p* < 0.05; ^††^statistically significant difference at *p* < 0.01; ^†††^statistically significant). The results were expressed as mean values ± SD.

## 4. Results and Discussion

The SLNs of voriconazole were projected to prepare by two different techniques, in order to check the influence of these techniques on physiochemical characteristics. SLN prepared by ultrasonication method relies on the dispersing technique in which sufficient high-energy input was necessary to break down the droplets into the nanometre range. It is predominantly effective in breaking up aggregates and in reducing the size and decreasing the polydispersity of nanoparticles which offers advantages, that is, easy handling, no critical parameters, and fast production process [23, 24]. Another method for formulating SLN, that is, microemulsion technique in which there is addition of a microemulsion to water, leads to precipitation of the lipid phase forming finer particles [25]. Low mechanical energy input, easy handling, and so forth are certain advantages of microemulsion method [23].

### 4.1. Particle Size, Polydispersity Index, and Zeta Potential Analysis

#### 4.1.1. Particle Size and Polydispersity Index Analysis

The results of particle size of freshly prepared lyophilized VCZ loaded formulations are depicted in Table 2 which are formulated with 2 different techniques. All SLN formulations showed a mean particle size below 400 nm which is an optimum size for ophthalmic delivery as the human eye can tolerate particles smaller than 10 *μ*m [14]. The particle size of formulations, that is, VUSLN-1 to VUSLN-5 ranges from 234 ± 3.52 nm to 288 ± 4.58 nm with ultrasonication method and VMSLN-6 to VMSLN-10 ranges from 308 ± 3.52 nm to 343 ± 3.51 nm with microemulsion technique respectively. The particle size increases with increase in polymer concentration were basically due to increased viscosity of dispersed phase resulting in larger nanodroplets formation. Similar findings were observed in earlier studies on Eudragit RL 100 based nanoparticulate system [20]. The particle size of SLNs prepared with microemulsion technique (VMSLN-6 to VMSLN-10) was significantly *p* < 0.01 higher as compared to mean particle size of formulations (VUSLN-1 to VUSLN-5) prepared with the ultrasonication method. This was explained by the fact that ultrasonication method generally leads to the breakdown of particles into smaller droplets during the preparation process.

#### 4.1.2. Polydispersity Index

The polydispersity index (PDI) is a marker of particle size distribution. Its value in case of submicron particles ranges from 0.15 to 0.3 indicates size homogeneity, while PDI greater than 0.3 results in heterogeneity [26]. The polydispersity index of all SLNs was significantly *p* < 0.05 varying from 0.188 ± 0.013 to 0.337 ± 0.015 as depicted in Table 2 indicating narrow size distribution which reveals the higher stability of solid lipid nanoparticles. Similar findings were reported in earlier studies on cyclosporine A incorporated cationic solid lipid nanoparticles for ocular drug delivery [27].

#### 4.1.3. Zeta Potential Analysis

Zeta potential is an important surface characterization technique which helps in determining the potential stability and surface charge of nanoparticulate system. Usually absolute large negative or positive zeta potential value required for colloidal dispersion stability as electrostatic repulsion between particles with same charges avoids aggregation [28]. All formulations of exhibited negative zeta potential values because of the presence of stearic acid (0.3% w/v), which were significantly *p* < 0.01, vary from −22.71 ± 0.63 mV to −28.86 ± 0.58 mV as depicted in Table 2 which is closer to −30 mV ensuring physical stability. Similar results have been reported in earlier studies on lopinavir solid lipid nanoparticles prepared by microemulsion technique [29].

### 4.2. Entrapment Efficiency (EE%)

The corresponding percent entrapment efficiency of SLN was found to be satisfactory high which is ranging from 61.91 ± 2.04% to 84.25 ± 1.11% (VUSLNs) prepared by ultrasonication method and 59.13 ± 1.97% to 72.50 ± 2.05% (VMSLNs) prepared by microemulsion technique, respectively, as depicted in Table 2. The results suggested that as the polymer concentration increases, the drug entrapment efficiency increased significantly (*p* < 0.01) due to its higher viscosity. But further increase in polymer concentration (VUSLN-5 and VMSLN-10 containing higher concentration of polymer as compared to others) showed decrease in entrapment efficiency which is basically due to decrease in drug loading, as entrapment efficiency is the ratio of actual drug loading and theoretical drug loading [14]. Higher entrapment efficiency of SLNs prepared with ultrasonication method is due to lesser particle size as compared to microemulsion technique.

### 4.3. Fourier Transform Infrared Spectroscopy (FTIR) Analysis

**Figure 1:**
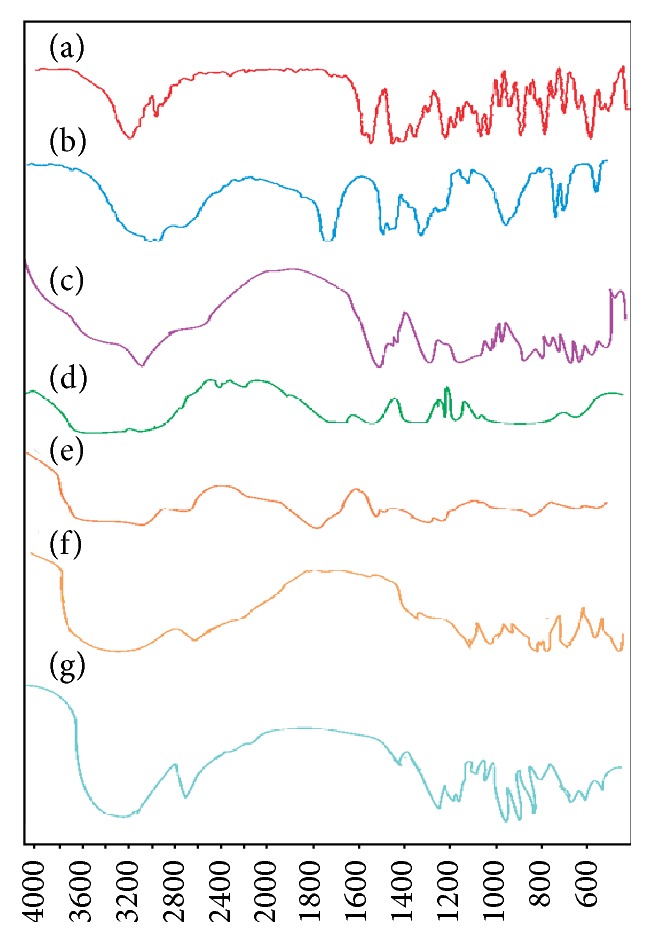
FTIR spectrum of (a) voriconazole, (b) stearic acid, (c) voriconazole, stearic acid, and Carbopol physical mixture, (d) Carbopol 934, (e) mannitol, (f) VUSLN-4, and (g) VMSLN-9.

FTIR is the backup analysis for the authentication of stability of crystalline shield of SLNs.  Figure 1 shows stacked IR spectra of pure drug, lipid, polymer, and physical mixtures and the prepared lyophilized VCZ loaded SLNs. The voriconazole FTIR spectrum showed OH stretching at 3200.09–3046.04 cm^−1^, C-N stretching at 1510.28–1451.28 cm^−1^, and C-F stretching at 1587.44–1451.28 cm^−1^, respectively. Stearic acid showed IR absorption band of C=O stretching at 1.700 cm^−1^. Spectrum of Carbopol 934 exhibited OH stretching at 3000–2950 cm^−1^ and the prominent peak between 1750–1700 cm^−1^ corresponds to carbonyl C=O stretching band. The spectrum of mannitol exhibited OH stretching at 3400 cm^−1^, OH in plane bending at 1420 cm^−1^, C-O stretching at 1081 cm^−1^, and OH out of plane bending for alcohol at 701 cm^−1^. The physical mixture of voriconazole, Carbopol 934, and stearic acid showed no major shifting of any functional peaks between the spectra of drug, lipids, polymer, and its physical mixtures. Hence, it was indicated that there was no interaction between the drug, lipids, and polymer used. The IR spectra of lyophilized nanoparticles (VUSLN-4) showed the characteristic peaks of stearic acid at 1702 cm^−1^, Carbopol 934 at 2935 cm^−1^, and mannitol at 3389 cm^−1^ and 1086 cm^−1^ correspond to OH stretching and C-O stretching, respectively, prepared with ultrasonication method. The same results were observed in case of lyophilized nanoparticles of voriconazole prepared by microemulsion technique. The characteristics peaks of drug in both the methods of SLN were diminished when compared with pure drug peak at same wave number (3197.80 cm^−1^). This indicated that the drug is dispersed in lipid matrix in a microcrystalline form and also the prominent peak of polymer in both methods of SLN could not be located due to dilution effect of lipids. Similar findings were observed in earlier studies on gatifloxacin solid lipid nanoparticles for ocular drug delivery [9].

### 4.4. Electron Microscope Examination

**Figure 2:**
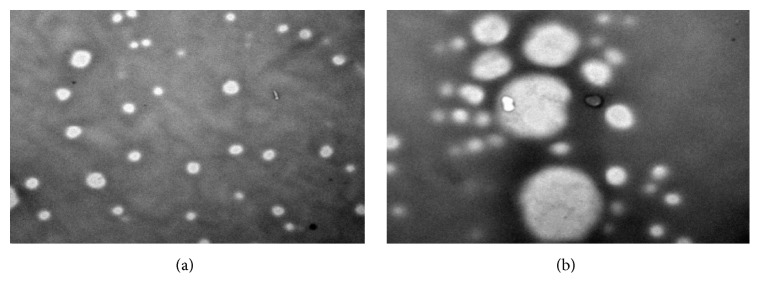
TEM images of (a) VUSLN-4 and (b) VMSLN-9.

Morphological examination of VCZ-SLN formulations was performed using transmission electron microscopy (TEM). TEM images of SLN formulations prepared by both methods are shown in (Figures 2(a) and 2(b)). This revealed that all prepared formulations were found to be spherical in shape with a smooth surface and suggesting possible stabilization of nanoparticles. These nanoparticles possibly do not cause any irritation to the ocular surface, as it is already reported that isometric particles with obtuse angles and edges cause less irritation than particles with sharp angles and edges [30]. There were minor differences observed in the general structure of the voriconazole SLN prepared by both methods. It may be confirmed by the fact that the shape of solid lipid nanoparticles is mainly decided by the property of lipid including solubility and film forming capability [31].

### 4.5. Powder X-Ray Diffraction (PXRD) Analysis

**Figure 3:**
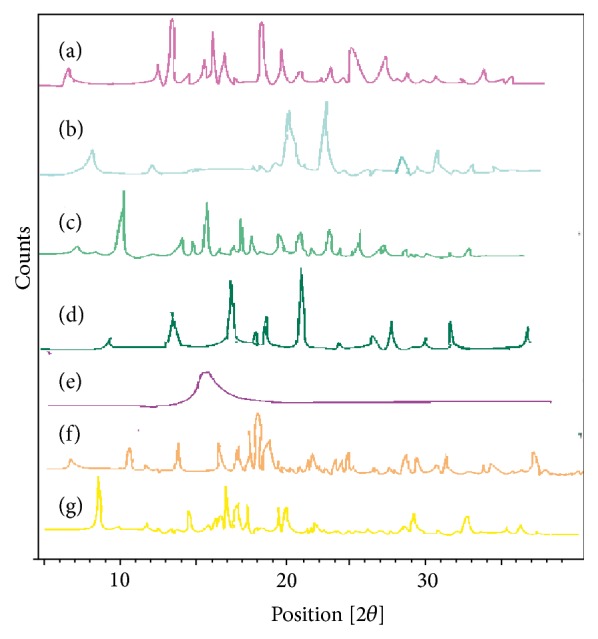
PXRD spectra of (a) voriconazole, (b) stearic acid, (c) voriconazole, stearic acid, and Carbopol physical mixture, (d) Carbopol 934, (e) mannitol, (f) VUSLN-4, and (g) VMSLN-9.

X-ray diffraction was carried out to evaluate the crystalline character of lipid matrices and VCZ-SLN prepared by both methods as shown in Figure 3 and displays the X-ray diffractograms of drug, lipids, polymer, physical mixture, mannitol, and lyophilized SLNs. Voriconazole exhibited the distinctive peaks at 12.13°, 13.45°, 14.20°, 19.30°, 21.14°, 27.15°, and 34.15° 2*θ*. The presence of sharp peaks in the diffractogram of voriconazole indicated its crystalline nature. From the XRD patterns of Carbopol 934, it is clear that the polymer is completely amorphous as there are no sharp peaks observed. The physical mixture of voriconazole, Carbopol 934, and stearic acid resulted in a relatively less crystalline form and exhibited peaks at 5.2°, 14.52°, and 20.28° 2*θ*. Mannitol showed crystalline nature and manifested several distinct peaks at 10.55°, 15.35°, 18.78°, 19.89°, 23.39°, 24.22°, 29.43°, 33.29°, 34.98°, and 39.53° 2*θ* which correspond to its *β* polymorphic form (mannitol exists in *α*, *β*, and *δ* polymorphic forms) [32]. The freeze-dried SLNs containing VCZ exhibited peaks at 8.94°, 9.76°, 15.98°, 19.70°, 20.42°, 20.54°, 21.44°, and 24.60° 2*θ* which appears to be contributed by mannitol and stearic acid. The intensity of peaks was sharpening and it may reveal that the SLN product is crystalline in nature [14, 18].

### 4.6. Differential Scanning Colorimetry (DSC) Analysis

**Figure 4:**
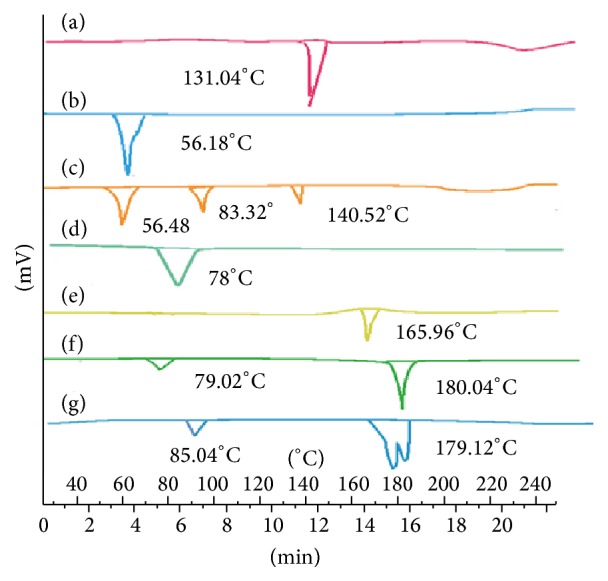
DSC of (a) voriconazole, (b) stearic acid, (c) voriconazole, stearic acid, and Carbopol physical mixture, (d) Carbopol 934, (e) mannitol, (f) VUSLN-4, and (g) VMSLN-9.

DSC is the technique to investigate the melting and recrystallization behaviour of the substance. The DSC thermograms of samples are represented in Figure 4. The thermogram of voriconazole is characterized by a sharp melting endotherm at 132.04°C and heat of fusion of 95.08 J/g. The thermal curve of stearic acid is characterized by a sharp melting endotherm at 56.18°C with heat of fusion of 157.19 J/g. The physical mixture of voriconazole, stearic acid, and Carbopol 934 showed characteristic peak at 140.52°C, 56.48°C, and 83.32°C, respectively with no depression in the melting point. A broad endothermic peak was observed at 78°C in the DSC scan of Carbopol 934. The thermal behaviour of mannitol is characterized by an endothermic peak at 165.96°C with heat of fusion of 291.31 J/g. The DSC curve of lyophilized SLNs showed a small endotherm at 81.02°C which corresponds to the melting point of Carbopol 934 followed by a sharp endotherm at 164.64°C which appears to be the depressed endothermic peak of *β* polymorph of mannitol. The thermograms did not show any melting peak of drug. This suggests that VCZ was present in amorphous state and drug is entirely entrapped in lipid matrix. As reported earlier, rapid quenching of microemulsion and presence of surfactant do not allow the drug to crystallize [33].

### 4.7. *In Vitro* Drug Release from VCZ-SLN

**Figure 5:**
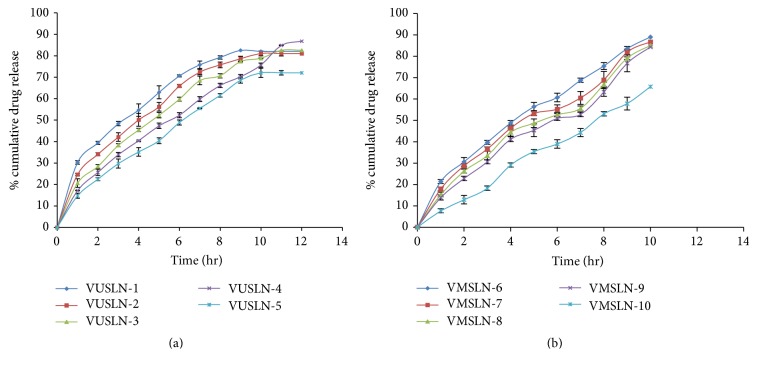
(a)* In vitro* release profile of voriconazole from SLN formulations prepared by ultrasonication method through dialysis membrane. (b)* In vitro* release profile of voriconazole from SLN formulations prepared by microemulsion technique through dialysis membrane.

*In vitro* release of VCZ from solid lipid nanoparticles prepared by both methods is represented in Figures 5(a) and 5(b). The drug release from SLN was assessed by dialysis membrane, using simulated tear fluid (pH 7.4) as the release medium. The solid nanoparticles made with ultrasonication method (VUSLN-4) showed 17.19 ± 0.09% drug release in 1 hr and 86.76 ± 0.78% drug release in 12 hr, while solid lipid nanoparticles prepared by microemulsion technique (VMSLN-9) demonstrated 13.86 ± 0.9% and 84.27 ± 1.01% drug release in 1 hr and 10 hr. There was statistically significant difference (*p* < 0.01) among both methods in drug release aspect. The results demonstrated that ultrasonication method caused a sustained drug release up to 12 h as indicated by the percentage drug release versus time profile (Figure 5(a)). The results suggested that entrapment of drug in solid lipid nanoparticles hinders the drug release, that is, initial burst release followed by slow release phase in case of ultrasonication method. This might be explained by the fact that smaller particle size formed by ultrasonication was responsible for increased surface area which increases the diffusional path length. Moreover, in case of microemulsion technique, the entrapment efficiency was lesser which ultimately affects the drug release. The polymer concentration increases the drug release more sustained, but in VUSLN-5 and VMSLN-10 the drug release is lesser due to improper drug loading [18]. SLNs prepared with ultrasonication method (VUSLN-4) were able to control the drug release till 12 hr, while solid lipid nanoparticles with microemulsion technique observed controlled drug release till 10 hr. Therefore, ultrasonication method was better as compared to microemulsion technique as it increases the precorneal residence time for longer period and provides sustained drug release.

### 4.8. Release Kinetics

The release data obtained was fitted into various kinetic models like zero-order, first-order, Higuchi, Korsmeyer-Peppas, and Hixson-Crowell model as depicted in Table 3 in order to determine the release mechanism and regression coefficients (*R* ^2^) [34]. The release of voriconazole from solid lipid nanoparticles fitted best in Higuchi-square-root release kinetics, which can be confirmed by comparing values for the regression coefficients of zero-order, first-order, Higuchi, Korsmeyer-Peppas, and Hixson-Crowell model (Table 3). The value of “*n*” (>0.45 to <0.89) and the diffusion exponent of Korsmeyer-Peppas equation confirmed that release of voriconazole from solid lipid nanoparticles is anomalous, that is, contributed by combination of dissolution and diffusion. Similar findings were observed in earlier studies on Eudragit RL 100 based nanoparticulate system of aceclofenac [18].

### 4.9. Corneal Permeation Studies

**Figure 6:**
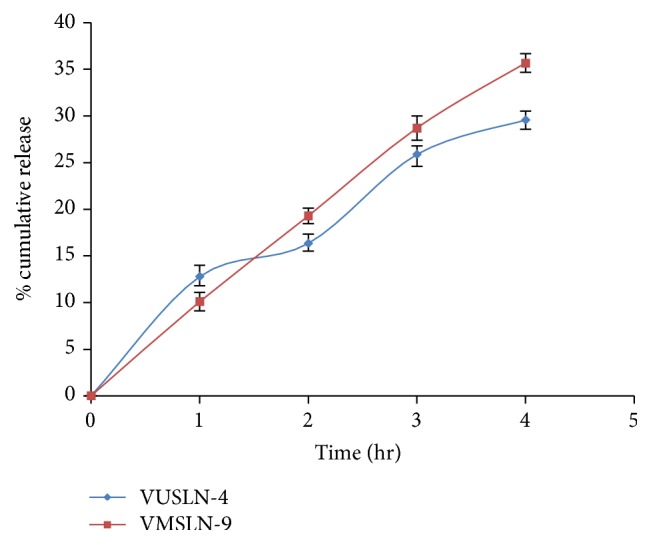
Transcorneal permeation profile of voriconazole from optimized SLN formulation through excised goat cornea.

*Ex vivo* transcorneal permeation studies compared the corneal permeation characteristics of the solid lipid nanoparticle formulation. The optimized batches (VUSLN-4 and VMSLN-9) prepared by 2 different methods according to* in vitro* release profile were selected for corneal permeation studies. The* in vitro* drug permeation studies of solid lipid nanoparticles of voriconazole through excised goat corneas were depicted in Figure 6. To imitate the real life conditions, excised goat corneas were used for permeation studies and the experiment was conducted for 4 hr considering cornea viability. The drug permeation from SLN prepared by ultrasonication method (VMSLN-4) was 29.56 ± 0.9%, which was less than the permeation observed with the (VMSLN-9) which was 35.65 ± 0.75% at the end of 4 h. The results suggested that SLN prepared by ultrasonication method demonstrated more sustained drug release which was possibly due to high entrapment efficiency in comparison to microemulsion technique.

Cornea (made of epithelium (lipophilic), stroma (hydrophilic), and endothelium (less lipophilic than epithelium)) acts as a lipophilic-hydrophilic barrier and the drug will have to partition through the barrier for corneal permeation while dialysis membrane filter acts as a mechanical barrier to drug diffusion. Accordingly, penetration through the excised goat cornea would be lower compared to that across the dialysis membrane.

### 4.10. Corneal Hydration Studies

The corneal hydration level of normal mammalian cornea is between 75% and 80% [32]. The increase in polymer concentration, in solid lipid nanoparticles, did not show any corneal damage. Corneal hydration value of the formulations prepared by both methods was between 76 and 79%, which indicated that the formulations did not cause any damage to the corneal tissue.

### 4.11. Stability Assessment

**Figure 7:**
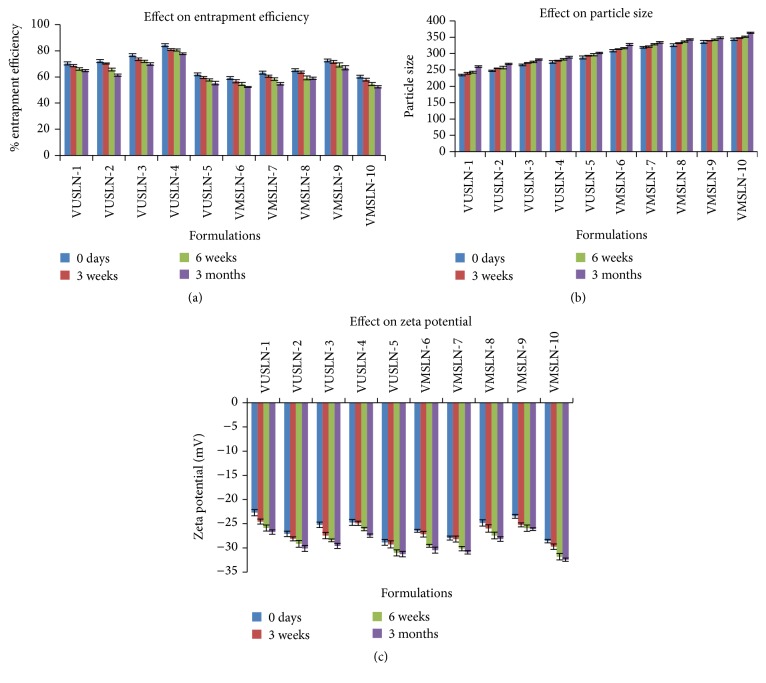
Stability profile of voriconazole loaded SLNs under room temperature. (a) Effect on particle size. (b) Effect on entrapment efficiency. (c) Effect on zeta potential. Mean ± SD (*n* = 3).

All the SLN formulations were kept on storage for three months at 25°C ± 2°C and 60% RH ±5% resulted in increase in particle size (Figure 7(a)). Increase in particle size ranges from 234 ± 1.5 nm to 301 ± 2.06 nm with batches prepared with ultrasonication method and from 308.66 ± 3.5 nm to 363.4 ± 1.25 nm with microemulsion technique. Entrapment efficiencies of SLN formulations were lowered by 6%–10% prepared by both methods after 3-month storage at room temp as shown in Figure 7(b) [17]. All the formulations showed slight increase in zeta potential ranging from 3.2% to 4.4% during room temperature storage conditions as depicted in Figure 7(c) [9]. The degradation of voriconazole loaded SLNs followed first-order kinetics. The degradation constant (*K*), *t*_90_ values, and shelf life of all the formulations at room temperature are shown in Table 4. The degradation rate constants (*K*) and shelf life (*t*_90_) at room temperature for all SLN formulations range from 1.08 × 10^−4^ day^−1^ to 1.62 × 10^−4^ day^−1^ and 644.11 to 963.8 days, respectively, as depicted in Table 4. The calculated *t*_90_ of optimized formulation (VUSLN-4) at room temperature was found to be 963.81 days confirming that the SLN would provide more than 2-year shelf life at room temperature [18].

## 5. Conclusion

The present investigation finally concluded that the lipophilic drugs like voriconazole can also be effectively incorporated in solid lipid (stearic acid) using tween 80 as stabilizer. The study was also able to explore the potential of both methods of preparation of voriconazole loaded solid lipid nanoparticles, that is, ultrasonication method and microemulsion technique. During the preparation of SLN, Carbopol 934 was used as controlled release agent. Increasing the concentration of the polymer (Carbopol 934) results in more sustained drug release of SLN due to formation of strong matrices due to its highly cross-linked structure. In determination of particle size study, the SLNs were obtained below 400 nm for all formulations with good PDI and negative zeta potential with optimum physiochemical characteristics. The negative zeta potential and fine particle size help to prolong precorneal residence time. The PXRD and DSC indicated decrease of drug crystallinity in the nanoparticles.* In vitro* release and* ex vivo* corneal permeation of drug from SLNs were found promising without causing any significant effect on the corneal hydration level. The nanoparticle was found to provide biphasic release pattern initial burst release followed by sustained release which fitted best into Higuchi-square-root release kinetics. The results demonstrated that SLN (VUSLN-4) prepared by ultrasonication method was able to sustain the drug release for up to 12 hours as compared to microemulsion technique. The optimized formulation (VUSLN-4) would provide more than 2-year shelf life at room temperature. However, the resulting solid lipid nanoparticles seem to be promising for providing a solution to the challenge caused by unsuccessful ocular delivery.

## Figures and Tables

**Table 1:** Composition of voriconazole loaded solid lipid nanoparticles.

Batches	Drug (% w/v)	Stearic acid (% w/v)	Tween 80 (mL)	Carbopol 934 (gm)	Dichloromethane (mL)	Distilled water (mL)
VUSLN-1	0.02	0.3	2	25	2	q.s
VUSLN-2	0.02	0.3	2	50	2	q.s
VUSLN-3	0.02	0.3	2	75	2	q.s
VUSLN-4	0.02	0.3	2	100	2	q.s
VUSLN-5	0.02	0.3	2	150	2	q.s
VMSLN-6	0.02	0.3	2	25	2	q.s
VMSLN-7	0.02	0.3	2	50	2	q.s
VMSLN-8	0.02	0.3	2	75	2	q.s
VMSLN-9	0.02	0.3	2	100	2	q.s
VMSLN-10	0.02	0.3	2	150	2	q.s

**Table 2:** Physiochemical characterization of VCZ loaded solid lipid nanoparticles.

S. number	Batches	Particle size (nm ± SD)	Zeta potential (mV ± SD)	^*∗*^PDI (±SD)	Entrapment efficiency (% ± SD)
1	^*∗*^VUSLN-1	234 ± 1.52	−22.71 ± 0.63	0.327 ± 0.01	70.26 ± 0.09
2	^*∗*^VUSLN-2	247 ± 2.08^††^	−27.10 ± 0.55^††^	0.192 ± 0.02^††^	72.08 ± 0.02^††^
3	^*∗*^VUSLN-3	265 ± 1.50^††^	−25.23 ± 0.56^††^	0.261 ± 0.07^†^	76.64 ± 0.04^††^
4	^*∗*^VUSLN-4	274 ± 4.04^††^	−24.73 ± 0.61^††^	0.291 ± 0.03^†^	84.25 ± 0.11^††^
5	^*∗*^VUSLN-5	288 ± 4.58^††^	−28.86 ± 0.58^††^	0.232 ± 0.05^†^	61.91 ± 0.04^††^
6	^*∗*^VUSLN-6	308 ± 3.51	−26.46 ± 0.30	0.248 ± 0.04	59.13 ± 0.07^††^
7	^*∗*^VUSLN-7	319 ± 2.64^†^	−27.96 ± 0.41^††^	0.188 ± 0.02^†^	63.23 ± 0.12^††^
8	^*∗*^VUSLN-8	325 ± 4.16^††^	−24.83 ± 0.65^††^	0.337 ± 0.06^†^	65.13 ± 0.05^††^
9	^*∗*^VUSLN-9	335 ± 4.50^††^	−23.46 ± 0.40^††^	0.219 ± 0.02^†^	72.50 ± 0.05^††^
10	^*∗*^VUSLN-10	343 ± 3.51^††^	−28.6 ± 0.36^††^	0.263 ± 0.02^†^	60.17 ± 0.05^††^

^*∗*^VUSLN: voriconazole loaded solid lipid nanoparticle prepared with ultrasonication method. ^*∗*^VMSLN: voriconazole loaded solid lipid nanoparticle prepared with microemulsion technique. ^*∗*^PDI: polydispersity index. ^*∗*^Values are mean ±  SD (*n* = 3).

^†^Statistically significant difference at *p* < 0.05; ^††^statistically significant difference at *p* < 0.01, from control (VUSLN-4 and VMSLN-9) as determined by one-way ANOVA followed by Dunnett's test.

**Table 3:** Kinetic profiles of *in vitro* drug release from solid lipid nanoparticles through dialysis membrane prepared by different techniques.

Batches	Zero-order model		First-order model		Higuchi model		Korsmeyer-Peppas model			Hixson-Crowell model	
	*R* ^2^	*K*_0_	*R* ^2^	*K*_1_	*R* ^2^	*K*_H_	*R* ^2^	*n*	*K*_KP_	*R* ^2^	*K*_HC_
VUSLN-1	0.8875	0.1075	0.9882	−0.0011	0.9925	3.0573	0.9957	0.4559	0.6995	0.9871	−0.0026
VUSLN-2	0.9276	0.1092	0.9891	−0.001	0.9917	3.2493	0.9944	0.5081	0.4820	0.9909	−0.0026
VUSLN-3	0.9526	0.1044	0.9852	−0.001	0.9919	3.3457	0.9949	0.5718	0.2854	0.9922	−0.0026
VUSLN-4	0.9773	0.1013	0.9323	−0.001	0.9793	3.4423	0.9923	0.6257	0.110	0.9678	−0.0027
VUSLN-5	0.9789	0.1004	0.9650	−0.0008	0.9720	3.1781	0.9885	0.6537	0.0072	0.9786	−0.0022
VMSLN-6	0.9632	0.1189	0.9245	−0.0013	0.9285	3.6661	0.9992	0.5843	0.2800	0.9640	−0.0032
VMSLN-7	0.9589	0.1145	0.8832	−0.0011	0.9581	3.5647	0.9824	0.6181	0.1620	0.9279	−0.0029
VMSLN-8	0.9601	0.1127	0.8738	−0.0011	0.9449	3.5609	0.9762	0.6635	0.020	0.9171	−0.0028
VMSLN-9	0.9653	0.1119	0.8609	−0.0010	0.9371	3.5845	0.9778	0.7037	0.1072	0.9078	−0.0028
VMSLN-10	0.9908	0.0945	0.960	−0.0007	0.9667	3.1574	0.9906	0.8909	0.7185	0.9744	−0.002

**Table 4:** Degradation rate constant and shelf life of SLN formulations after 3-month storage.

Batches	Degradation constant (*K*) in day^−1^	*t*_90_ (days)	Shelf life (years)
VUSLN-1	1.37 × 10^−4^	763.25	2.09
VUSLN-2	1.59 × 10^−4^	659.88	1.80
VUSLN-3	1.33 × 10^−4^	783.99	2.14
VUSLN-4	1.08 × 10^−4^	963.81	2.64
VUSLN-5	1.29 × 10^−4^	811.54	2.22
VMSLN-6	1.32 × 10^−4^	789.91	2.16
VMSLN-7	1.30 × 10^−4^	803.77	2.20
VMSLN-8	1.42 × 10^−4^	737.14	2.01
VMSLN-9	1.26 × 10^−4^	826.98	2.26
VMSLN-10	1.63 × 10^−4^	644.11	1.76
